# Association between triglyceride to high-density lipoprotein cholesterol ratio and type 2 diabetes risk in Japanese

**DOI:** 10.1038/s41598-022-25585-5

**Published:** 2023-03-06

**Authors:** Huijuan Wang, Changming Wang, Xiuping Xuan, Zhouni Xie, Yuanyuan Qiu, Huiping Qin, Zhong Xiaoning

**Affiliations:** 1 Department of Respiratory, The First Affiliated Hospital, Guangxi Medical University, 6 Shuangyong Road, Nanning, 530021 Guangxi People’s Republic of China; 2 Department of General Medicine, Guilin People’s Hospital, The Fifth Affiliated Hospital of Guilin Medical University, Guilin, 541002 People’s Republic of China; 3 Department of Respiratory Medicine, Guilin People’s Hospital, The Fifth Affiliated Hospital of Guilin Medical University, Guilin, 541002 People’s Republic of China; 4 Department of Endocrinology, The First Affiliated Hospital of Guangxi Medical University, 6 Shuangyong Road, Nanning, Guangxi 530021 People’s Republic of China

**Keywords:** Endocrinology, Risk factors

## Abstract

Abnormal lipid metabolism is known to increases the risk for metabolic diseases, such as type 2 diabetes mellitus(T2DM). The relationship between baseline ratio of triglyceride to HDL cholesterol (TG/HDL-C) and T2DM in Japanese adults was investigated in this study. Our secondary analysis included 8419 male and 7034 female Japanese subjects who were free of diabetes at baseline. The correlation between baseline TG/HDL-C and T2DM was analyzed by a proportional risk regression model, the nonlinear correlation between baseline TG/HDL-C and T2DM was analyzed by a generalized additive model (GAM), and the threshold effect analysis was performed by a segmented regression model. We conducted subgroup analyses in different populations. During the median 5.39 years follow-up, 373 participants, 286 males and 87 females, developed diabetes mellitus. After full adjustment for confounders, the baseline TG/HDL-C ratio positively correlated with the risk of diabetes (hazard ratio 1.19, 95% confidence interval 1.09–1.3), and smoothed curve fitting and two-stage linear regression analysis revealed a J-shaped relationship between baseline TG/HDL-C and T2DM. The inflection point for baseline TG/HDL-C was 0.35. baseline TG/HDL-C > 0.35 was positively associated with the development of T2DM (hazard ratio 1.2, 95% confidence interval 1.10–1.31). Subgroup analysis showed no significant differences in the effect between TG/HDL-C and T2DM in different populations. A J-shaped relationship was observed between baseline TG/HDL-C and T2DM risk in the Japanese population. When TG/HDL-C was higher than 0.35, there was a positive relationship between baseline TG/HDL-C and the incidence of diabetes mellitus.

## Introduction

Diabetes is a chronic disease that seriously affects human health and has a widespread global impact, it has become a growing public health problem worldwide and it has been recognized as a global public health challenge^1^. According to an estimate provided by the International Diabetes Federation (IDF), by 2021, 537 million adults are living with diabetes, and this number is expected to rise to 643 million by 2030 and to 783 million by 2045^2^. In Japan, the prevalence of diabetes has been increasing dramatically since 1997, especially in males^3^. Many Asian countries, including China, India, Singapore, and Japan, have a considerable prevalence of diabetes, and the prevalence of diabetes has increased extremely rapidly in these regions in recent years^3^. The prevalence of diabetes, primarily type 2 diabetes, poses enormous social and economic problems that may hinder national and global development. Many factors such as unhealthy diet, obesity, and a sedentary lifestyle are thought to contribute to T2DM^4^. Given the global economic and social burden of diabetes, understanding the risk factors for diabetes that can be intervened to enhance diabetes prevention can help reduce the economic burden on countries and individuals. Active and effective prevention of diabetes mellitus can lead to early detection of diabetic patients, facilitate timely and effective treatment, reduce, and delay the occurrence and development of diabetic complications, improve the quality of life of patients, reduce the disability rate and prolong life expectancy.

The pathogenesis of diabetes mellitus is very complex, and as research continues, it is found that diabetes mellitus is the result of multiple factors and mechanisms acting together. Abnormal lipid metabolism is both an important factor in the development of diabetes mellitus and an important cause of its complications^5,6^. Diabetes is characterized by insulin resistance (IR), insufficient insulin secretion and increased hepatic glucose output^7,8^. IR is a condition in which the efficiency of insulin in promoting glucose uptake and utilization by cells is reduced for various reasons, i.e., the biological effects produced by insulin do not work properly^9^. The gold standard for assessing β-cell function and insulin sensitivity is the hyperinsulinemic euglycemic clamp technique^10^. However, this technique is more challenging to apply in clinical practice due to its inconvenience and high cost, so other simple biomarkers that reflect IR or β-cell dysfunction may be beneficial for screening for diabetic diabetes. Disorders of lipid metabolism, including elevated serum triglyceride levels and decreased serum high-density lipoprotein cholesterol, among others. Lipid metabolism disorders plays a crucial role in the pathogenesis of diabetes^11^. Previous studies have shown that the ratio of triglycerides to HDL cholesterol (TG/HDL-C) is strongly associated with insulin resistance, and it is expected to be a simple and easy predictor of IR^12–15^. However, despite the potential of the TG/HDL-C ratio as a simple and easy metric to predict diabetes risk, there are very limited reports of prospective cohort studies investigating the relationship between baseline TG/HDL-C ratio and T2DM risk. Although studies on the relationship between TG/HDL-C and T2DM have only been reported in Chinese Singaporean, Korean, Chinese, and Iranian populations^13,16–19^, there have been few studies on data from the Japanese population. To the best of our knowledge, one study data from Ibaraki-Prefecture, Japan, showed that the TG/HDL-C ratio was positively associated with incident diabetes^20^; However, their study did not clarify the linear/nonlinear effect of TG/HDL-C on diabetes risk and the appropriate cutoff value.

In our present investigation, we re-analyze the data from the previously published study by Okamura et al.^21^. TG/HDL-C was utilized as an independent variable in the secondary analysis, and the outcome variables and other covariates were the same as in the original study.

## Methods

### Data source

Information of participants in this study was acquired from the NAGALA database. The data package for this study was collected, organized by Okamura et al. and submitted to the Dryad database for free use by the researchers. We conducted a secondary analysis of this dataset^22^. Variables included in this dataset: age, gender, body mass index (BMI), weight, waist circumference (WC), high-density lipoprotein cholesterol (HDL-C), γ-glutamyl transpeptidase (GGT), triglycerides (TG), total cholesterol (TC), hemoglobin A1c (HbA1c), diastolic blood pressure (DBP), systolic blood pressure (SBP), alanine aminotransferase (ALT), fasting plasma glucose (FPG), ethanol consumption, aspartate aminotransferase (AST), smoking status, alcohol consumption, follow-up time, fatty liver, diabetes mellitus, and exercise habits.

### Study participants

NAGALA is a longitudinal cohort study in the Gifu Area of Japan analyzing^21^. The study project collected data from participants in a health checkup program at Murakami Memorial Hospital in Japan^21^. In this medical checkup program, 60% of the participants received one or two medical checkups per year^21^. Previous researchers recruited 20,944 participants from individuals who participated in medical checkup programs from 2004 to 2015. Participants with the following conditions at baseline will be excluded: Missing relevant data (including exercise, alcohol consumption, height, HDL-cholesterol, and abdominal ultrasound), viral /alcoholic hepatitis, alcohol abuse, impaired fasting blood glucose, diabetes, or Any medication used at baseline examination. The final 15,453 subjects were eligible for our study. Because previous studies were submitted to the ethics committee of Murakami Memorial Hospital for approval^21^, the current study is exempt from ethical review.

### Variables measurement and definitions

As previously mentioned^21^, the researchers used questionnaires, physical examinations, and blood tests to obtain baseline data from the participants. Subjects were categorized depending on average weekly ethanol and type of alcohol intake. Alcohol intake of less than 40 g per week is defined as no or minimal alcohol consumption^23^. Weekly alcohol intake of 40 g to 140 g is defined as light alcohol consumption^23^. Alcohol intake of 140 g to 280 g per week is defined as moderate alcohol consumption^23^. Weekly alcohol intake greater than 280 g is defined as heavy alcohol consumption^23^. Participants were divided into non-smokers, ex-smokers, and current smokers based on their smoking status at baseline. Non-smokers were defifined as participants who never smoked cigarettes, ex-smokers as participants who had smoked in the past but who quit smoking until the baseline visit, and current-smokers as participants who smoked at the baseline visit^21^.

Regular participation in sports > 1x/week is defined as regular exercise. ^24^. Body mass index is calculated as the number of kilograms of body weight divided by the square of the number of meters of height^25^. The ratio of TG/HDL-C was measured by dividing the fasting triglyceride level (mmol/L) into the fasting High-density lipoprotein cholesterol level (mmol/L). Gastroenterologists diagnose fatty liver by reviewing abdominal ultrasound based on four known criteria (liver brightness, liver, and kidney echo contrast, vascular blurring, and depth attenuation)^26^.

### Definition of T2DM

Participant self-report ,FPG ≥ 7 mmol/L, or HbA1c ≥ 6.5%,were used to identify incident type 2 diabetes^21,27^.

### Statistical analysis

Frequencies or percentages was used to express categorical variables. The mean ± standard deviation was used to represent normally distributed continuous variables, and the median (*P*25, *P*75) is used to represent skewed continuous variables. One-way ANOVA, Kruskal–Wallis H-test, and chi-square test were used to compare the differences between groups. Univariate regression models were used to examine the effect of each variable on T2DM. The covariates found to be significantly different in the univariate analysis were screened for confounding factors. Multivariate Cox proportional risk models were used to examine the risk prediction of exposure variables on outcome variables, and the risk ratios (HRs) with 95% confidence intervals (CIs) were estimated to assess the risk of the outcome variables. The confounding factors screened included: sex, age, alcohol consumption, exercise habits, smoking status, fatty liver, BMI, fasting plasma glucose, total cholesterol, and HbA1c. We show three models: unadjusted analysis model (model 1); partially adjusted analysis (model 2): adjusted for sex and age only; and fully adjusted analysis (model 3), adjusted for all screened confounders. Researchers used a generalized additive model (GAM, restricted cubic spline function) to examine if there was a nonlinear relationship between baseline TG/HDL-C and the risk of T2DM. Threshold effects were evaluated by smoothed curve fitting and segmented regression models. A stratified logistic regression model was used to perform subgroup analyses based ages, genders, exercise habits, and smoking status. The likelihood ratio test was used to test the interactions among subgroups. We used R version 3.4.3 and Empower (R) version 2.0 to perform statistical analysis of the study data. *P* < 0.05(bilateral) is the criterion for significance.

### Ethical approval

Approval of the research protocol: The data comes from the public database. In the previously published article^21^. Takuro Okamura et al. has clearly stated that: the study was approved by the ethics committee of Murakami Memorial Hospital.

## Result

### Participants' baseline characteristics

In our study, 15,453 individuals free of diabetes at baseline were included. The mean age of the subjects was 43.71 ± 8.90 years, and 45.52% of the subjects were females.

The median follow-up time was 5.39 years. During this period, 373 individuals developed diabetes, 87 females and 286 males. The prevalence of diabetes was 2.4%. Table 1 shows the baseline characteristics of subjects grouped according to quartiles of TG/HDL-C ratio. In the group with higher TG/HDL-C, participants were older and had higher BMI, weight, waist circumference and blood pressure (SBP and DBP). In the presence of elevated TG/HDL-C quartiles, ALT, GGT, FPG and TC gradually increased. There were considerably more smokers and heavy drinkers in the group Q4 than in the other three groups (Q1-Q3). The proportion of those with fatty liver and diabetes increased with increasing TG/HDL-C.

**Table 1:** Baseline characteristics of participants by categories of the baseline TG/HDL-C in the NAGALA study, 2004–2015.

Variable	TG/HDL-C quartiles				P-value
	Q1 (0.21 ± 0.06)	Q2 (0.39 ± 0.06)	Q3 (0.68 ± 0.11)	Q4 (1.68 ± 0.98)	
Participants (n)	3699	3967	3916	3871	
Age, year	41.18 ± 8.39	43.34 ± 8.85	44.97 ± 9.06	45.23 ± 8.68	< 0.001
Ethanol consumption, g/week	1.00 (0.00–22.00)	1.00 (0.00–60.00)	2.80 (0.00–84.00)	12.00 (1.00–90.00)	< 0.001
BMI, kg/m^2^	20.26 ± 2.28	21.33 ± 2.64	22.54 ± 2.93	24.27 ± 3.07	< 0.001
WC, cm	70.51 ± 6.86	73.85 ± 7.92	77.94 ± 8.19	83.36 ± 7.96	< 0.001
ALT, IU/L	14.00 (11.00–17.00)	15.00 (12.00–20.00)	18.00 (14.00–23.00)	23.00 (17.00–32.00)	< 0.001
AST, IU/L	16.00 (13.00–20.00)	17.00 (14.00–20.00)	17.00 (14.00–21.00)	19.00 (16.00–24.00)	< 0.001
GGT, IU/L	12.00 (10.00–15.50)	13.00 (11.00–18.00)	16.00 (12.00–23.00)	22.00 (16.00–33.00)	< 0.001
HDL-C, mmol/L	1.85 ± 0.38	1.58 ± 0.29	1.35 ± 0.25	1.09 ± 0.21	< 0.001
TC, mmol/L	4.86 ± 0.80	5.01 ± 0.81	5.17 ± 0.86	5.45 ± 0.87	< 0.001
TG, mmol/L	0.38 ± 0.12	0.62 ± 0.13	0.91 ± 0.20	1.73 ± 0.78	< 0.001
HbA1c, %	5.15 ± 0.29	5.14 ± 0.31	5.18 ± 0.33	5.21 ± 0.34	< 0.001
FPG, mmol/L	4.97 ± 0.39	5.10 ± 0.40	5.22 ± 0.39	5.35 ± 0.37	< 0.001
SBP, mmHg	108.32 ± 12.99	111.94 ± 14.07	116.35 ± 14.70	121.13 ± 14.88	< 0.001
DBP, mmHg	66.95 ± 9.21	69.67 ± 9.82	72.96 ± 10.17	76.56 ± 10.25	< 0.001
**Gender**					< 0.001
Female	2838 (76.72%)	2241 (56.49%)	1380 (35.24%)	575 (14.85%)	
Male	861 (23.28%)	1726 (43.51%)	2536 (64.76%)	3296 (85.15%)	
**Fatty liver**					< 0.001
No	3624 (97.97%)	3686 (92.92%)	3208 (81.92%)	2198 (56.78%)	
Yes	75 (2.03%)	281 (7.08%)	708 (18.08%)	1673 (43.22%)	
**WC ≥ 90 in men, ≥ 80 in women**					< 0.001
No	3489 (94.32%)	3617 (91.18%)	3360 (85.80%)	2975 (76.85%)	
Yes	210 (5.68%)	350 (8.82%)	556 (14.20%)	896 (23.15%)	
**Habit of exercise**					< 0.001
Yes	3002 (81.16%)	3258 (82.13%)	3207 (81.89%)	3280 (84.73%)	
No	697 (18.84%)	709 (17.87%)	709 (18.11%)	591 (15.27%)	
**Alcohol consumption**					< 0.001
Non	3135 (84.75%)	3085 (77.77%)	2901 (74.08%)	2681 (69.26%)	
Light	300 (8.11%)	444 (11.19%)	496 (12.67%)	514 (13.28%)	
Moderate	211 (5.70%)	323 (8.14%)	363 (9.27%)	460 (11.88%)	
Heavy	53 (1.43%)	115 (2.90%)	156 (3.98%)	216 (5.58%)	
**Smoking status**					< 0.001
Never	2918 (78.89%)	2611 (65.82%)	2012 (51.38%)	1486 (38.39%)	
Past	452 (12.22%)	680 (17.14%)	858 (21.91%)	959 (24.77%)	
Current	329 (8.89%)	676 (17.04%)	1046 (26.71%)	1426 (36.84%)	
**Incident diabetes**					< 0.001
No	3677 (99.41%)	3921 (98.84%)	3833 (97.88%)	3649 (94.27%)	
Yes	22 (0.59%)	46 (1.16%)	83 (2.12%)	222 (5.73%)	

Data were mean ± SD or median (P25–P75)/N (%) for skewed variables or numbers (proportions) for categorical variables.

*BMI* body mass index, *WC* waist circumference, *ALT* alanine aminotransferase, *AST* aspartate aminotransferase, *GGT* γ-glutamyl transferase, *HDL-C* high-density lipoprotein cholesterol, *TC* total cholesterol, *TG* triglyceride, *HbA1c* hemoglobin A1c, *FPG* fasting plasma glucose, *SBP* systolic blood pressure, *DBP* diastolic blood pressure.

### Univariate analysis

We performed the univariate analysis of the relationship of each variable with T2DM, and the results are shown in Table 2. Without adjusting for other variables, all of the covariables, except light and moderate ethanol consumption, were related to the occurrence of T2DM; of these, exercise habits and HDL-C were negatively related to the onset of diabetes, and the other variables were positively associated with diabetes. The study also observed that males had a greater to acquire diabetes than females, that subjects with fatty liver were at significantly higher risk of acquiring T2DM than subjects without fatty liver, and that subjects with thicker waist circumference (WC ≥ 90 in males and WC ≥ 80 in females) had a stronger chance of developing T2DM, and those current and former smokers were more likely to develop diabetes than never smokers. Heavy drinkers have a higher risk of developing T2DM than those who do no or minimal alcohol consumption.

**Table 2:** Univariate analysis for incident diabetes.

Covariate	Statistics	OR (95%CI)	P-value
**Gender**			
Female	7034 (45.52%)	Reference	
Male	8419 (54.48%)	2.81 (2.20, 3.58)	< 0.0001
Age, year	43.71 ± 8.90	1.04 (1.03, 1.05)	< 0.0001
Ethanol consumption, g/week	47.71 ± 82.31	1.00 (1.00, 1.00)	0.0001
**Fatty liver**			
No	12,716 (82.29%)	Reference	
Yes	2737 (17.71%)	7.43 (6.02, 9.18)	< 0.0001
BMI, kg/m^2^	22.12 ± 3.13	1.26 (1.23, 1.29)	< 0.0001
WC, cm	76.47 ± 9.11	1.10 (1.09, 1.11)	< 0.0001
**WC ≥ 90 in men, ≥ 80 in women**			
No	13,441 (86.98%)	Reference	
Yes	2012 (13.02%)	4.09 (3.29, 5.07)	< 0.0001
**Baseline BMI ≥ 25**			
No	12,932 (83.69%)	Reference	
Yes	2521 (16.31%)	4.64 (3.77, 5.71)	< 0.0001
ALT, IU/L	19.99 ± 14.35	1.03 (1.02, 1.03)	< 0.0001
AST, IU/L	18.40 ± 8.64	1.03 (1.02, 1.04)	< 0.0001
Body weight, kg	60.63 ± 11.62	1.06 (1.05, 1.07)	< 0.0001
**Habit of exercise**			
No	12,747 (82.49%)	Reference	
Yes	2706 (17.51%)	0.74 (0.55, 1.00)	0.0491
GGT, IU/L	20.31 ± 18.14	1.01 (1.01, 1.02)	< 0.0001
HDL-C, mmol/L	1.46 ± 0.40	0.10 (0.07, 0.14)	< 0.0001
TC, mmol/L	5.13 ± 0.86	1.47 (1.32, 1.64)	< 0.0001
TG, mmol/L	0.91 ± 0.66	2.00 (1.83, 2.19)	< 0.0001
HbA1c, %	5.17 ± 0.32	36.72 (26.06, 51.73)	< 0.0001
**Alcohol consumption**			
Non	11,802 (76.37%)	Reference	
Light	1754 (11.35%)	1.01 (0.72, 1.42)	0.9441
Moderate	1357 (8.78%)	1.22 (0.86, 1.72)	0.2722
Heavy	540 (3.49%)	2.55 (1.73, 3.76)	< 0.0001
**Smoking status**			
Never	9027 (58.42%)	Reference	
Past	2949 (19.08%)	1.64 (1.24, 2.17)	0.0005
Current	3477 (22.50%)	2.78 (2.21, 3.50)	< 0.0001
FPG, mmol/L	5.16 ± 0.41	24.58 (17.91, 33.74)	< 0.0001
SBP, mmHg	114.49 ± 14.97	1.03 (1.02, 1.04)	< 0.0001
DBP, mmHg	71.58 ± 10.50	1.05 (1.04, 1.06)	< 0.0001
TG (mmol/L)/HDL-C(mmol/L)	0.74 ± 0.75	1.75 (1.62, 1.89)	< 0.0001

*CI* confidence interval, *OR* odds ratio, *BMI* body mass index, *WC* waist circumference, *ALT* alanine aminotransferase, *AST* aspartate aminotransferase, *GGT* γ-glutamyl transferase, *HDL-C* high-density lipoprotein cholesterol, *TC* total cholesterol, *TG* triglyceride, *HbA1c* hemoglobin A1c, *FPG* fasting plasma glucose, *SBP* systolic blood pressure, *DBP* diastolic blood pressure.

### Independent effect of baseline TG/HDL-C on the risk of T2DM

A Cox proportional risk regression model was used to analyze the association with baseline TG/HDL-C and T2DM risk. Table 3 shows the results of the analyses in the no-adjusted, partially adjusted, and fully adjusted models, respectively. When TG/HDL-C was used as a continuous variable, it was strongly related to diabetes risk in the unadjusted model, with each 1-unit increase in TG/HDL-C associated with a 46% increase in diabetes risk. After partially adjustment and full adjustment for confounding, a positive association between TG/HDL-C and T2DM risk remained. We then categorized participants into quartiles based on baseline TG/HDL-C. The results showed that the relationship between TG/HDL-C and T2DM first decreased and then increased in model 3. HRs and 95%CIs for Q2–Q4 were 0.90 (0.54–1.51), 0.86 (0.52–1.42), and 1.12 (0.68–1.84), respectively, when compared to Q1.

**Table 3:** Relationship between TG/HDL-C and incident diabetes.

Outcome	Model 1		Model 2		Model 3	
	HR (95% CI)	*P*-value	HR (95% CI)	*P*-value	HR (95% CI)	*P*-value
TG/HDL-C	1.46 (1.40, 1.52)	< 0.001	1.41 (1.35, 1.48)	< 0.001	1.19 (1.09, 1.30)	< 0.001
**TG/HDL-C(quartile)**						
Q1	Reference		Reference		Reference	
Q2	1.61 (0.97, 2.68)	0.0646	1.41 (0.84, 2.34)	0.1926	0.90 (0.54, 1.51)	0.6869
Q3	2.90 (1.81, 4.64)	< 0.0001	2.22 (1.37, 3.61)	0.0013	0.86 (0.52, 1.42)	0.5549
Q4	7.59 (4.90, 11.77)	< 0.0001	5.51 (3.45, 8.80)	< 0.0001	1.12 (0.68, 1.84)	0.6593

Model 1: not adjusted other covariants.

Model 2: adjusted for gender and age.

Model 3: adjusted for gender, age, exercise habits, body mass index, hemoglobin A1c, fatty liver, total cholesterol, smoking situation, alcohol consumption, fasting plasma glucose.

*HR* hazard ratio, *CI* confidence interval, *TG* triglyceride, *HDL-C* high-density lipoprotein cholesterol.

### Threshold effect analysis

**Figure 1:**
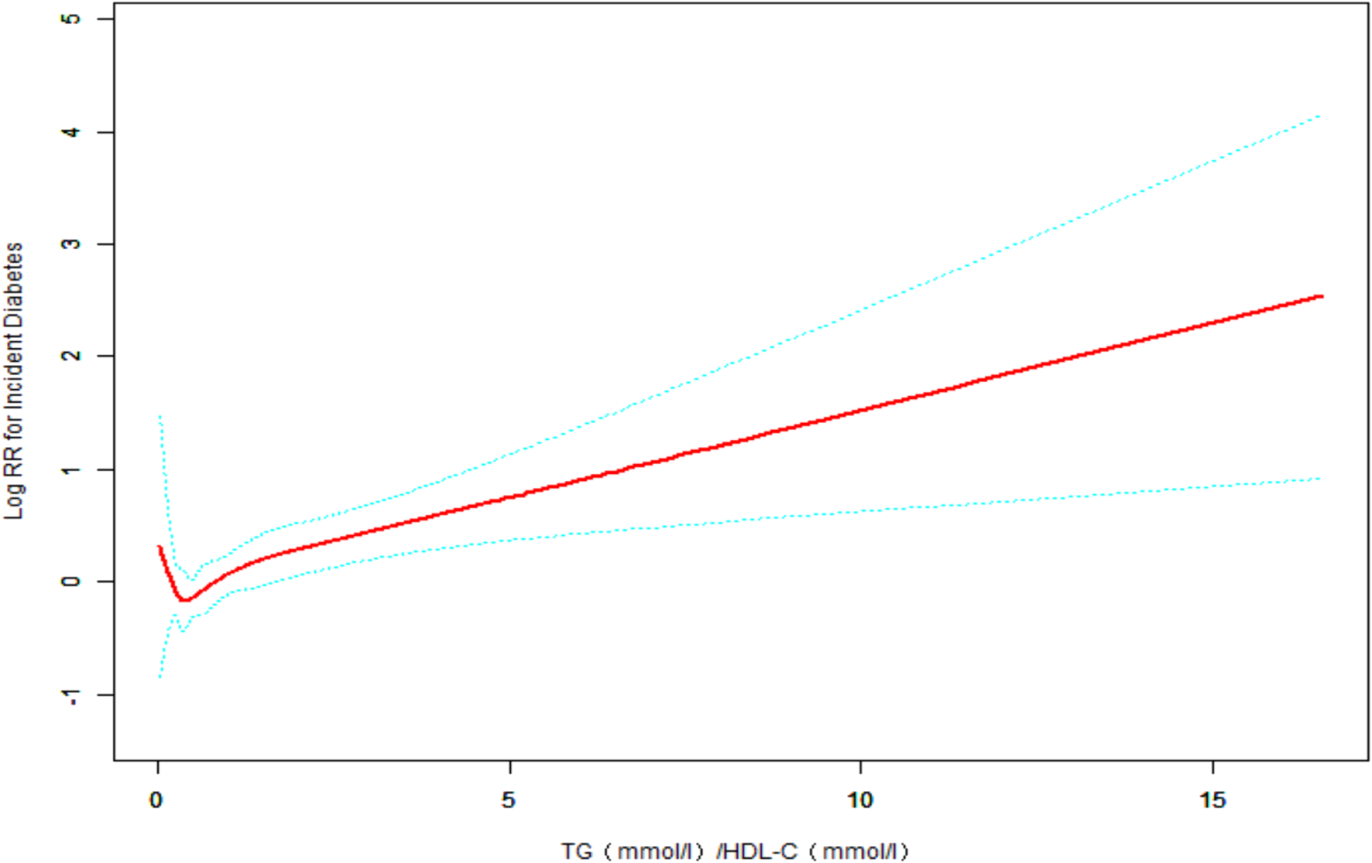
GAM and smoothed curve fitting were used to investigate the relationship between TG/HDL-C ratio and the incidence of T2DM. The red solid line indicates the estimated risk of developing T2DM. The green dashed line indicates the 95% confidence interval of the fit. After adjusting for gender, age, exercise habits, body mass index, fatty liver, total cholesterol, hemoglobinA1c, smoking status, alcohol consumption, fasting plasma glucose, a J-shaped relationship was detected between TG/HDL-C ratio and the incidence of T2DM, with the risk of developing T2DM decreasing with increasing TG/HDL-C on the left side of the inflection point and the opposite relationship observed on the right side of the inflection point. Association between TG/HDL-C and T2DM in the Japanese population: J-shaped association between TG/HDL-C and T2DM. The solid red line indicates the smoothed curve fit between the variables. The green dashed line indicates the 95% confidence interval of the fit. Adjusted for gender, age, exercise habits, body mass index, fatty liver, total cholesterol, hemoglobinA1c, smoking status, alcohol consumption, fasting plasma glucose.

As Cox regression analysis showed inconsistent results on the prevalence for diabetes when TG/HDL-C was considered as a categorical and continuous variable. The nonlinear relationship between TG/HDL-C and T2DM was analyzed using the generalized additive model (GAM). A J-shaped association of TG/HDL-C with T2DM was found through smoothed curve fitting (Fig. 1).

Threshold effects analysis was performed by smoothed curve fitting and segmented regression models to determine the inflection point of the association of TG/HDL-C with T2DM. The inflection point of TG/HDLC was 0.35. TG/HDL-C < 0.35, TG/HDL-C was negatively associated with T2DM, whereas TG/HDL-C > 0.35, TG/HDL-C was positively associated with T2DM (Table 4).

**Table 4:** The results two-piecewise linear regression mode.

Outcomes	HR (95% CI)	*P*-value
One-line linear regression model	1.19 (1.09, 1.30)	< 0.001
**Inflection point of TG/HDL-C**		
< 0.35	0.27 (0.01, 6.55),	0.4234
> 0.35	1.20 (1.10, 1.31),	< 0.0001
Log-likelihood ratio test		0.0376

Adjusted for gender, age, exercise habits, body mass index, hemoglobin A1c, fatty liver, total cholesterol, smoking situation, alcohol consumption, fasting plasma glucose.

*HR* hazard ratio, *CI* confidence interval, *TG* triglyceride, *HDL-C* high-density lipoprotein cholesterol.

### The results of subgroup analyses

The correlation between T2DM and TG/HDL-C in different subgroups was shown in Table 5. We grouped the variables of age, gender, smoking status, exercise habits, and alcohol consumption and performed subgroup and interaction analyses, respectively. The results showed that the correlation between TG/HDL-C and T2DM was stable across subgroups, and further interaction analysis did not reveal any significant differences between subgroups.

**Table 5:** Subgroup analyses of the association between TG/HDL-C and incident type 2 diabetes.

Characteristic	No. of participants	HR (95%CI)	*P* -value	*P* for interaction
**Age (year)**				0.8626
18–35	3091	1.24 (0.93, 1.64)	0.1408	
36–39	2493	1.29 (1.09, 1.53)	0.0028	
40–44	3281	1.19 (1.01, 1.40)	0.0398	
45–51	3373	1.15 (0.96, 1.37)	0.1314	
52–79	3215	1.15 (0.96, 1.37)	0.1314	
**Gender**				0.6478
Female	7034	1.26 (0.99, 1.61)	0.0629	
Male	8419	1.19 (1.08, 1.30)	0.0003	
**Alcohol consumption**				0.6951
Non	11,802	1.17 (1.05, 1.30)	0.0033	
Light	1754	1.28 (1.04, 1.57)	0.0204	
Moderate	1357	1.35 (0.78, 1.54)	0.0384	
Heavy	540	1.10 (1.10, 1.30)	0.5894	
**Fatty liver**				0.8435
No	12,716	1.19 (1.03, 1.38)	0.0171	
Yes	2737	1.21 (1.08, 1.34)	0.0005	
**Smoking status**				0.6490
Never	9027	1.07 (0.90, 1.28)	0.4385	
Past	2949	1.32 (1.11, 1.56)	0.0015	
Current	3477	1.22 (1.07, 1.40)	0.0033	
**Habit of exercise**				0.5374
No	12,747	1.21 (1.10, 1.33)	< 0.0001	
Yes	2706	1.11 (0.86, 1.44)	0.4247	

Adjusted for age, gender, fatty liver, BMI, habit of exercise, total cholesterol, HbA1c, alcohol consumption, smoking status, fasting plasma glucose except the subgroup variable.

## Discussion

This report explored the relationship between TG/HDLC and T2DM. The correlation between TG/HLD-C and T2DM has been investigated in several studies on populations in different regions of China, and their studies^12,13^ showed a non-linear connection between TG/HDL-C and diabetes events. This is consistent with our finding. Unexpectedly, in this study, T2DM showed a J-shaped relationship with baseline TG/HDL-C after adjusting for confounders gender, age, exercise habits, BMI, fatty liver, total cholesterol, FPG, HbA1c, alcohol consumption, and smoking status (Table 4 and Fig. 1). In addition, we calculated a threshold value of 0.35 for TG/HDL-C by threshold effect analysis. It's worth noticing that the link between TG/HDL-C and T2DM had the opposite effect on different sides of threshold value. Participants had the lowest risk of T2DM when TG/HDL-C was approximately 0.35, and when TG/HDL-C was lower than 0.35, TG/HDL-C was negatively linked with T2DM. However, the risk was not statistically significant (HR 0.27, 95% CI 0.01–6.55, *P*-value 0.4234). When TG/HDL-C > 0.35, there was a positive association between TG/HDL-C and the risk of diabetes, suggesting that the risk of diabetes is increased with either a high or low TG/HDL-C. The mechanism by which a high TG/HDL-C ratio increases the incidence of diabetes is unclear. Previous studies have suggested dyslipidemia as a causal factor of insulin resistance^28^. An increase in TG and decreased HDL-C levels through genetic variants in lipid-related genes could cause insulin resistance^29^. It results in compensatory hyperinsulinemia, leading to aggravation of hypertriglyceridemia. However, the J-shaped association between TG/HDL-C and T2DM and the mechanisms behind the threshold value are unclear. The issue has significant physiological and clinical implications based on the impact of dyslipidemia on diabetes.

According to studies, disorders of lipid metabolism have a major role in the development of T2DM, and the impact of dyslipidemia on developing type 2 diabetes cannot be ignored^5,30–32^. Elevated triglycerides decrease insulin sensitivity and increase the risk of developing diabetes^33–35^, while high-density lipoprotein cholesterol plays a protective role^36^. Elevated plasma triglycerides and decreased HDL-C are danger markers and predictors of diabetic events and insulin resistance in the population^37^. It was shown that elevated triglycerides, TG/HDL-C and decreased HDL-C can contribute to the onset and progression of diabetes^38^. The ratio of TG/HDL-C is a relatively easy, convenient, and low-cost indicator obtained during routine clinical care or physical examination and is considered by some authors to be a highly sensitive and specific predictive indicator of diabetes^31,38^. TG/HDL-C is better than TG or HDL-C alone on predicting diabetes risk^38^. Therefore, it is more than recommended to use TG/HDL-C for predicting impaired beta-cell and insulin resistance^39–43^. Studies have reported that the predictive function of TG/HDL-C for diabetes is race-specific, and it has been suggested that TG/HDL-C could be a useful predictive indicator for diabetes in Chinese Singaporean, Hispanic, and African American, as well as Chinese populations^16,17,41,44^. We reviewed the relevant literature and found several studies that associated TG/HDL-C with T2DM. Liu's research team and Kim's research team noted a close connection between the TG/HDL-C and T2DM^13,19^. A cohort study of 114,787 Chinese participants showed a positive relationship between TG/HDL-C and diabetes risk, using subjects in the lowest quartile of TG/HDL-C as a reference, subjects in the highest quartile of TG/HDL-C were more susceptible to acquiring T2DM^13^. Similar results were found by Uruska et al. for the study of the TG/HDL-C ratio to assess IR in patients with type 1 diabetes^45^. Our results also show that TG/HDL-C is positively associated with the risk of T2DM by a proportional hazards model, and their relationship remains positive after adjusting for different confounders, and the results suggest an independent relationship between them.

Glucose and lipid metabolism are influenced by various factors, and it remains controversial whether TG/HDL-C was correlation with diabetes differs between genders.Liu et al. showed that the results of subgroup analysis indicated that the correlation of TG/HDL-C ratio on the incidence of T2DM was not significantly different between genders, with a *P*-value of 0.53 for their interaction^12^. Similarly, a cohort study by Chen et al. showed the same results for gender-specific subgroups, with P = 0.058 for their interaction^13^. To investigate the differences between Japanese men and Japanese women in the association of TG/HDL-C withT2DM, we performed a subgroup analysis in this study (Table 5). Our study showed a non-significant difference in TG/HDL-C and T2DM between genders, female (HR = 1.26, *P* = 0.06) vs. male (HR = 1.19, P < 0.01), with a *P*-value of 0.65 for the interaction.

Our findings show there was no significant difference in the risk of diabetes with increased TG/HDL-C by gender in the Japanese population. And the finding indicated with increased TG/HDL-C that the risk of diabetes was consistent between genders in the Japanese population. However, other studies have obtained different results: for example, some studies in Iranian, Chinese, Chinese Singaporeans, and Japanese populations suggest that the correlation between TG/HDL-C and T2DM as significantly higher in females than in males^17,19,20,46^. Another study concluded that high TG/HDL-C was an influential factor in incident diabetes in men participants and that TG/HDL-C was available to infer the risk of T2DM in male, but their study did not include female participants^47^. Qin et al. investigated the effect of TG/HDL-C on diabetes in Chinese adults and whether there were differences between genders and discovered that the correlation between TG/HDL-C and diabetes was independent^44^. This association was significant only in Chinese adult males^44^. Similarly, Zhang et al. reported that the trajectory of TG/HDL-C was only observed to be correlated with the progression of diabetes in men, but not in women^16^. Whether gender affects the relationship between lipid metabolism and diabetes can be further investigated. We have analysed these studies that are inconsistent with our results and speculate that the reasons for the different results may be due to the following factors: Firstly, it is thought that the higher risk of diabetes in females than in males is due to the dysregulation of glucose and lipid metabolism caused by the decline in oestrogen levels in women after menopause^48^, which may put women at greater risk of developing T2DM. The mean age of the women in our study population was 43.25 years and our findings are limited by the fact that the original data did not register whether the women were menopausal or not. Second, Song S et al. thought that differences in dietary patterns were responsible for gender differences in the effect of TG/HDL-C on the risk of diabetes in Korean adults^49^. Third, the association between TG/HDL-C and insulin resistance is race-specific, and gender differences may vary between races.

Our study has several strengths. First, this investigation is the first to show a J-shaped correlated between baseline TG/HDL-C and T2DM risk. Second, this is a large cohort study, involving a relatively large number of people, and is highly representative of the Japanese population. Third, to improve the stability of the results, we analyzed TG/HDL-C as categorical and continuous variables, respectively. Fourth, to explore the impact of baseline TG/HDL-C on T2DM in different populations, a subgroup analysis was conducted in this study.

Despite its strengths, the study has some limitations. First, some covariates were not available in the study due to the limitations of the original study data; therefore, residual confounding may be present in this study. Second, this investigation did not distinguish between types of diabetes mellitus. But, type 1 diabetes is not extremely common in the Japanese population^50^. Therefore, we inferred that almost all new-onset diabetes in this study was T2DM. Third, in this investigation, oral glucose tolerance tests were not used to screen for T2DM, so the findings may have underestimated the risk of developing T2DM. Fourth, given that T2DM prevalence is associated with race and region, and the study population in this study was Japanese, this result is not necessarily generalizable to populations outside of Japan.

## Conclusion

Our findings indicate an independent relationship between baseline TG/HDL-C and T2DM in the Japanese population. This investigation is the first to show a J-shaped relationship between baseline TG/HDL-C and T2DM risk. People with TG/HDL-C higher than 0.35 are at greater risk of developing diabetes, and we should pay more attention to this group for diabetes prevention.

## Data Availability

All data are available in the "DATADRYAD" database (https://doi.org/10.5061/dryad.8q0p192).
